# Prevalence and Predictors of Burnout in Midwives: A Systematic Review and Meta-Analysis

**DOI:** 10.3390/ijerph17020641

**Published:** 2020-01-19

**Authors:** Nora Suleiman-Martos, Luis Albendín-García, José L. Gómez-Urquiza, Keyla Vargas-Román, Lucia Ramirez-Baena, Elena Ortega-Campos, Emilia I. De La Fuente-Solana

**Affiliations:** 1 Nursing Department, Faculty of Health Sciences, University of Granada, Campus Universitario de Ceuta, C/Cortadura del Valle SN, 51001 Ceuta, Spain; norasm@ugr.es; 2 Andalusian Health Service, Avenida del Sur N. 11, 18014 Granada, Spain; lualbgar1979@ugr.es; 3 Nursing Department, Faculty of Health Sciences, University of Granada, Avenida de la Ilustración N. 60, 18016 Granada, Spain; jlgurquiza@ugr.es; 4 Faculty of Psychology, University of Granada, Campus Universitario de Cartuja SN, 18071 Granada, Spain; keyvarom@ugr.es; 5 Spanish Red Cross Nursing School, Universidad de Sevilla, Avda. de la Cruz Roja, 41009 Sevilla, Spain; 6 Faculty of Psychology, University of Almería, Carretera de Sacramento SN, 04120 Almería, Spain; elenaortega@ual.es; 7 Brain, Mind and Behaviour Research Center (CIMCYC), Faculty of Psychology, University of Granada, Campus Universitario de Cartuja SN, 18011 Granada, Spain; edfuente@ugr.es

**Keywords:** burnout, meta-analysis, midwife, predictors, systematic review

## Abstract

The prevalence of burnout in midwives has been briefly studied. Given the negative effects of burnout syndrome in the physical and mental health, and also related to the quality of care provided, rates of absenteeism and sick leave; identifying related factors for the syndrome are needed. The aim was to determine the prevalence, levels, and factors related to the burnout syndrome, measured with the Copenhagen Burnout Inventory in midwives. A systematic review and meta-analysis were selected from CINAHL, LILACS, ProQuest, PsycINFO, PubMed, SciELO, and Scopus databases, with the search equation “burnout AND (midwife OR midwives OR nurses midwives)”. Fourteen articles were found with a total of 8959 midwives. Most of the studies showed moderate levels of personal burnout. The prevalence obtained was 50% (95% CI = 38–63) for personal burnout; 40% (95% CI = 32–49) for work-related burnout; and 10% (95% CI = 7–13) for client-related burnout. Midwives’ age, less experience, and living alone constitute the main related factors, as well as, the scarcity of resources, work environment, and the care model used. Most midwives present personal and work-related burnout, which indicates a high risk of developing burnout. Personal factors and working conditions should be taken into account when assessing burnout risk profiles of midwives.

## 1. Introduction

The well-being of the healthcare workforce is related to levels of job satisfaction and motivation [1]. Its deterioration can provoke many disorders with the burnout syndrome being one of the most frequent. Burnout is a psychological syndrome characterized by physical, emotional, and mental fatigue, which appears as a result of exposure to a series of stressors in a chronic manner [2].

There are several validated tools for the measurement of burnout syndrome such as the Maslach Burnout Inventory (MBI) [3], the Professional Quality of Life (ProQOL) [4], or the Copenhagen Burnout Inventory (CBI) [5].

The MBI is one of the main measurement scales used in the literature, characterized by a three-dimensional concept (emotional exhaustion, depersonalization, and personal accomplishment), but there are some controversies between its dimensions [5]. That is why given the growing concerns about the methodological quality of the MBI, some authors developed the CBI to reflect more accurately the physical and mental exhaustion [6]. This instrument provides information according to source and causality, without introducing ambiguous concepts such as depersonalization and personal accomplishment.

The CBI consists of three subscales: Personal-burnout (degree of physical and mental exhaustion experienced by the individual), with six items; work-related burnout (degree of physical and psychological exhaustion related to the person’s work), with seven items; and client-related burnout (degree of physical and psychological exhaustion related to the person’s work with clients) with six items [6]. All items use a five-point scale score with a range between 0 (low burnout) to 100 (severe burnout). A score between 50–74 represents a moderate level of burnout, a score between 75–99 represents a high level of burnout, while a score of 100 represents severe burnout [5].

Midwives are continually exposed to stress-inducing factors associated with absenteeism and work leave profession due to the low degree of personal and professional satisfaction perceived [7,8]. Moreover, the closure of health units and the reorganization of services has reduced their autonomy and medicalized the assistance [9,10]. It supposes a negative impact related to the ability of concentration and communication skills and endangers the quality care [11].

To improve the relationship between mother–midwife, new models of care have appeared, such as the caseload midwifery [12]. This model is focused on the continuity of care, with a 24 h availability upon the mother’s needs, and a strong emotional link between mother and midwife [13]. Caseload model offers autonomy and independence in care, and is considered to be a related factor against burnout [14,15].

Many authors have studied the prevalence and levels of burnout in health professionals [16,17,18,19,20], and even some systematic reviews and meta-analyses have explored their relationship with possible related factors [21,22,23,24,25]. However, these papers have been performed in different hospital units, excluding maternity ward or did not distinguish between the nurses and midwives [26].

Although the multiple factors related to burnout syndrome in other groups may apply to midwives, only a few studies analyze its impact. Moreover, few studies have been developed through the use of a measurement tool of burnout syndrome. Therefore, the importance of this work is focused on the CBI scale, being an instrument validated in midwives with greater reliability [27].

Furthermore, it is important to clarify which are the burnout related variables to contribute to the service reorientation and care models and identify the prevalence among midwives. However, no known meta-analyses have been conducted regarding this context. Therefore, the objectives of this paper are: (1) To calculate a meta-analytical estimation of the prevalence of burnout syndrome in midwives, (2) to describe the levels of the three CBI subscales (personal, work-related, and client-related burnout), (3) to analyze the factors related to burnout syndrome.

## 2. Materials and Methods

### 2.1. Design

A systematic review and meta-analysis were performed following the PRISMA (Preferred Reporting Items for Systematic Reviews and Meta-analyses) guidelines [28] (Table S1).

### 2.2. Data Sources and Search Strategy

The following sources were consulted: CINAHL (Cumulative Index of Nursing and Allied Health Literature), LILACS (Latin American and Caribbean Health Sciences Literature), ProQuest (Proquest Health and Medical Complete), PsycINFO, PubMed, SciELO (Scientific Electronic Library Online), and Scopus. The search was conducted in December 2019, using the MeSH terms “burnout AND (midwife OR midwives OR nurses midwives)” as a search strategy.

### 2.3. Study Selection

**Figure 1:**
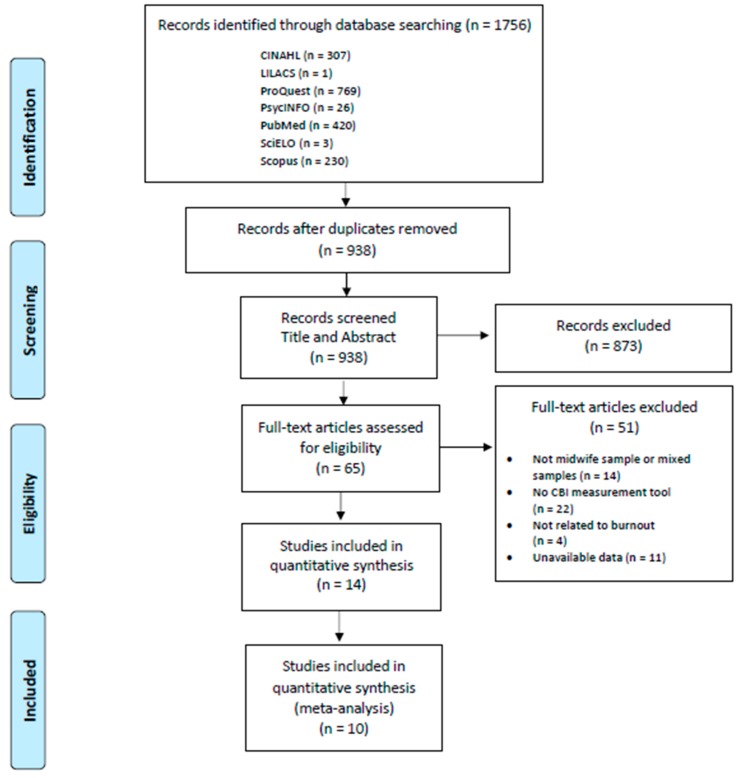
Flow diagram of the selection process.

First, two authors, after removing duplicate studies, independently reviewed the title and abstract of the articles found. A third author was consulted in case of disagreement. Subsequently, the full-text articles were reviewed, according to the inclusion criteria and a critical reading was done (see Figure 1).

### 2.4. Inclusion and Exclusion Criteria

The inclusion criteria were: (1) Primary quantitative studies, (2) sample of midwives, (3) the use of the CBI scale as a measurement instrument, (4) the measurements of burnout levels expressed in mean or percentage values, (5) written in English, Spanish, or French languages. Any date of publication was acceptable. We excluded articles that did not meet the following criteria: (1) Mixed samples lacking independent data on midwives, (2) not providing sufficient statistical information to calculate the effect size, (3) not using the CBI as a tool to measure burnout, (4) midwifery students sample.

### 2.5. Data Extraction

Two authors extracted data from all included studies using a data coding form. A third author verified the data in case of disagreement. The following variables were obtained for each of the articles: (1) Information about the study (authors, year of publication, country), (2) study design, (3) sample selection, (4) instrument reliability coefficient, (5) sample size, (6) burnout levels (mean, standard deviation), (7) percentages for each subscale of the CBI (personal, work, and client-related burnout), (8) factors related to burnout syndrome.

To access the reliability of the data coding by the researchers, the intraclass correlation coefficient was calculated and it was 0.97 (minimum = 0.96; maximum = 1). The Cohen Kappa coefficient was used for categorical variables and it was 0.96 (maximum = 0.97; maximum = 1).

### 2.6. Risk Assessment of Bias and Quality

The STROBE (Strengthening the Reporting of Observational Studies in Epidemiology) guide was used, proposed by Sanderson et al. [29]. The domains evaluated were: Selection bias, measurement bias, design specific bias, confounding bias, statistical method bias, and conflict of interest or funding source.

A quality assessment tool, Oxford Center for Evidence-Based Medicine Levels of Evidence Working Group (OCEBM) [30], was used for the level of evidence and grade of recommendation.

### 2.7. Data Synthesis

Three meta-analyses of randomized effects were performed, to calculate the prevalence of burnout and the corresponding confidence interval, one for each CBI subscale.

The program used was StatsDirect (StatsDirect Ltd., Cambridge, UK) for the analysis, presenting the results grouped on forest plots.

Data heterogeneity was assessed using the I^2^ index. This test measures the percentage of the variability in effect estimates that is due to heterogeneity. There was significant heterogeneity if the I^2^ values were greater than 50% [31]. Publication bias was assessed using the Egger lineal regression test.

## 3. Results

### 3.1. Literature Search Results

The initial search provided 1756 articles. After reading the title and abstract, 873 articles were not selected. After reading the full text of the remaining articles, a total of 14 articles were finally selected. The study selection process is shown in Figure 1.

### 3.2. Characteristics of the Study Sample

All of the included studies (n = 14) were cross-sectional. Half of the studies were conducted in Australia [32,33,34,35,36,37,38], two in Denmark [5,39], and the rest in Canada [40], New Zealand [34], Norway [41], Sweden [42], and the United Kingdom [43]. The total number of midwives was 8958. All of the studies, except one [5], were done after 2013. Most of the articles used convenience sampling, except two articles, being randomized [39,41]. The reliability of the CBI questionnaire estimated in nine articles was acceptable, with a Cronbach α minimum of 0.76 and maximum of 0.93 (Table 1). Regarding the methodological quality, all studies presented an adequate level of quality. The evaluation is represented in Table 1 and Table 2.

### 3.3. Mean Scores for Personal, Work, and Client-Related Burnout in Midwives

Moderate levels in personal-burnout are shown with average scores from 50 points to 65.4 [32,34,35,36,38,40,43,44]; although other authors found low levels of personal-burnout [5,33,37,39,42].

Regarding the work-related burnout dimension, two authors found moderate levels of burnout [36,43], while the rest of the authors established lower scores between 33.85 and 48.44 [38,42].

Finally, all the authors found low average scores in client-related burnout, from a minimum of 8.3, to a maximum of 38.4 [5,44]. This information is listed in Table 1.

### 3.4. Modifiable and Non-Modifiable Factors that Contribute to Burnout

Among the personal variables, a lower age range and being single is related to a higher burnout score [35,36,40,41,42,43]. The family plays a protective role [41], although having children generates controversy; for certain authors, this fact contributes to reducing personal and work-related burnout [35], others only found a relationship with client-related burnout [42,43], and for some, having children increases the levels [40], or even no relation was found [36].

Regarding the geographical area, studies carried out in northern Europe, show lower levels of burnout [5,39,41]. The postnatal area and performing education and management functions increase burnout [35,36], as well as, working in rural areas reduces the scores [35].

Regarding work-related variables, the autonomy and a major experience are positive related factors [35,36,37,41,42,43]. The lack of staff and resources [34,42], low salary [36], a poor professional recognition and organization [34,41], and a negative work environment [34,40,42], are considered factors related to burnout. This is related to high rates of dropout from the profession of up to 58.9% [42].

Moreover, other associated psychological variables were found, such as medium-high levels of anxiety (20%–38%), depression (17.3%–33%), and stress (22.1%–36.7%) [32,42,43].

### 3.5. Burnout Levels in the Different Care Models

The caseload midwifery model presents lower levels of burnout than the traditional models [33,34,35,37,39,44]. Some factors such as autonomy, care continuity, work schedule flexibility, and work for task organization, are the main aspects identified in the caseload midwifery model [34,37].

Despite showing a 24 h availability, the satisfaction levels are elevated, thus working a high number of hours, which is not related to a higher risk of burnout [41,42].

### 3.6. Meta-Analytical Prevalence Estimate

A total of 5946 midwives were included in this meta-analysis (Table 3). Egger linear regression shows an absence of publication bias, being for personal-burnout *p* = 0.30; for work-related burnout *p* = 0.44; and for client-related burnout *p* = 0.88.

Regarding the analysis of the heterogeneity of the studies, the I^2^ index was 98.5% for personal burnout, 97.3% for work-related burnout, and 90% for client-related burnout, with a high level of heterogeneity in the three subscales of burnout.

**Figure 2:**
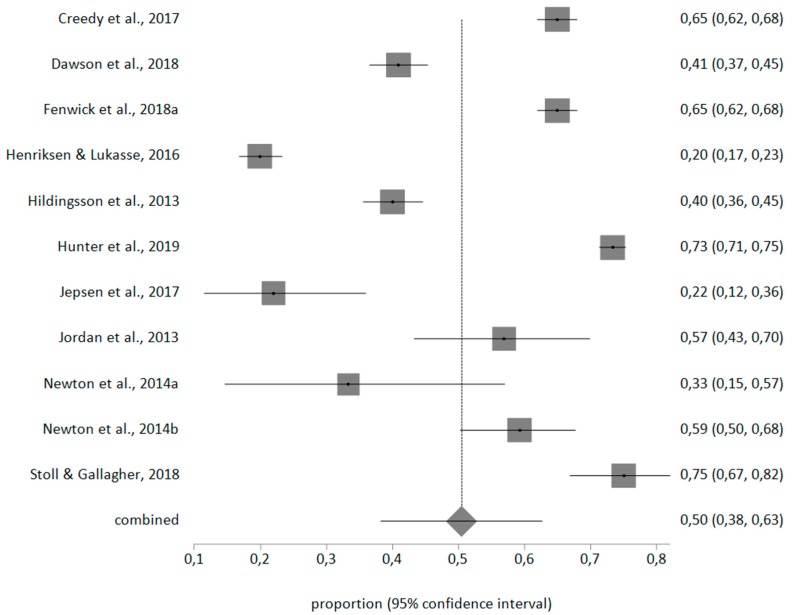
Forest plot of personal-related burnout prevalence.

**Figure 3:**
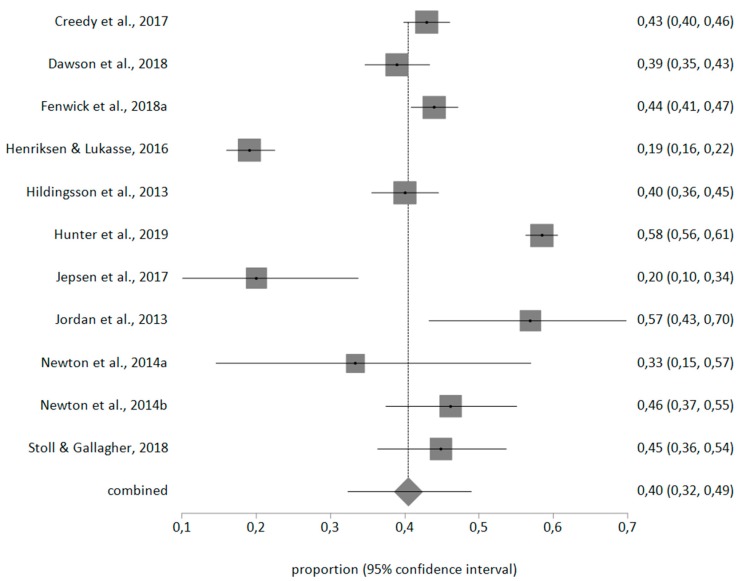
Forest plot of work-related burnout prevalence.

**Figure 4:**
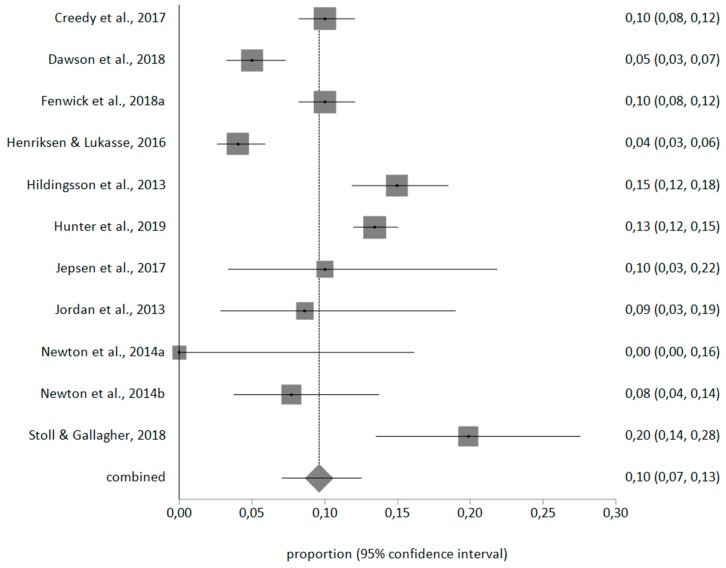
Forest plot of client-related burnout prevalence.

With a random effects meta-analysis, the prevalence for personal burnout was 50% (95% CI = 38–63), for work-related burnout was 40% (95% CI = 32–49), for client-related burnout was 10% (95% CI = 7–13). The meta-analytical estimate is shown in Figure 2, Figure 3 and Figure 4.

## 4. Discussion

The purpose of this systematic review and meta-analysis was to analyze burnout levels and prevalence in midwives who carry their work in any healthcare setting, as well as, the related factors that could influence the development of the syndrome.

There are high levels of personal and work-related burnout, and low levels of client-related burnout; however, other authors found high levels in work and client-related burnout dimensions [27]. The results show a prevalence for personal-burnout of 50%; for work-related burnout of 40%; and for client-related burnout of 10%, similar to other studies [45].

In relation to personal-burnout, the high prevalence is related to a low salary and a lack of professional recognition which could reduce the commitment at work [8,46,47,48,49].

The high prevalence found in work-related burnout is due to occupational variables, such as media shortages, labour disputes, and lack of organization [50]. Similarly to the results found in this review, many authors associate these factors to leave the profession up to 50% [8,42]. Although deep dissatisfaction with their role in the organisation is also related to the exposure to chronic stress and anxiety situations [51]. Even the fast and inadequate care information after discharge, putting in risk the mother and the newborn’s health, could be related to higher levels of burnout in the postnatal area [52]. A lower score is found in rural areas, although several studies report that in these areas there is a lack of resources and a high level of stress, that predispose to burnout appearance [53,54]. Moreover, other authors found that management and administration functions are associated with higher levels of burnout [55].

Although we found low levels of client-related burnout, other authors report high levels related to constant demands and family claims [27].

On the three subscales, the young, less experienced, and single midwives, presented higher levels of burnout [56,57], probably related to poor practical skills and lack of emotional support [58]. Family and having children are considered positive related factors; although the relationship of the latter case is not clear [23,50].

In caseload midwives, the number of working hours does not appear to increase the level of burnout; although in other health groups this relationship is found [59].

The benefits of the caseload model are clear. The fact that this model reduces burnout levels is due to a continuity of care and autonomy [1,15,60]. Therefore, it may be that in northern European countries where currently trialing caseload care mode and Australia that has already adopted this model, leading to score lower levels of burnout [14]. In addition, greater job satisfaction is found, since, despite being available at any time of the day, they can organize work and family life balance thanks to working schedule flexibility [61]. However, other authors found difficulties related to the high responsibility in care [62].

The burnout syndrome is a complex, subjective, and multifactorial term, so it is difficult to attribute its development to a specific cause. However, the measurement by the CBI is very useful. This is because the CBI addresses more realistically the levels of physical and mental exhaustion of health personnel, and in our case in midwives [27,63]. Moreover, this model distinguishes between occupational and personal related factors, and it is also interesting because it contemplates the relationship between healthcare professionals and patients [64].

The key to early prevention is the identification of risk factors and the reorganization of care [65]. Essential strategies are increasing work motivation and developing techniques to cope with the great physical and mental burden, to take account by healthcare administrators and managers [66].

This study presented some limitations. The first one, since they are cross-sectional studies, it is difficult to establish a causal relationship over time. Second, in the majority of the articles, a convenient sampling was used and this increases the risk of selection bias [31]. Moreover, the heterogeneity of the obtained data is due to the different geographical locations, where healthcare systems, structures, and resources vary depending on the economic status [67].

## 5. Conclusions

Midwives are vulnerable to the burnout syndrome because moderate levels in personal-burnout and high prevalence in personal and work-related burnout have been reported. The factors that appear to exert more influence are age, less experience, and living alone. Furthermore, some work-related positive variables are autonomy and continuity of care.

The use of the Copenhagen Burnout Inventory allows identifying the different contexts related to burnout, both at a personal and work-related level, establishing with more precision the origin of the cause. However, longitudinal studies are needed to determine the possible risk factors that could influence burnout levels in midwives.

Adopting new care models and reorganizing the system providing continuity in care, are aspects to be developed on sanitary organizations against burnout. Future research should develop strategies programs in midwives, aimed at reducing personal and work-related burnout.

## Figures and Tables

**Table 1:** Sample characteristics.

Studies	Design and Sample	Instrument (Cronbach α)	M (SD)			Main Results	EL	RG
			PB	WB	CB			
Creedy et al. [32], 2017, Australia	Cross-sectional Convenience sample N = 990	CBI (PB = 0.90 WB = 0.88 CB = 0.89) DASS	55.9 (18.06)	44.69 (19.23)	19.32 (19.22)	Depression PB (r = 0.62 *) WB (r = 0.63 *) CB (r = 0.39 *) Anxiety PB (r = 0.51 *) WB (r = 0.53 *) CB (r = 0.31) Stress PB (r = 0.59 *) WB (r = 0.63 *) CB (r = 0.39 *)	2c	B
Dawson et al. [33], 2018, Australia	Cross-sectional Convenience sample N1 = 99 caseload midwives N2 = 402 standard care	CBI MPQ	N1 = 39.84 (18.8) N2 = 45.7 (19.6)	N1 = 36.6 (19.9) N2 = 46.3 (20.2)	N1 = 17.9 (18.7) N2 = 18.3 (16.8)	N1 vs. N2 PB: *p* = 0.007; 95% CI (1.59–10.17) WB: *p* < 0.001; 95% CI (5.29–14.12) CB: *p* = 0.82; 95% CI (−3.34–4.23)	2c	B
Dixon et al. [34], 2017, New Zealand	Cross-sectional Convenience sample N1 = 473 caseload midwives N2 = 452 employed N3 = 148 both	CBI (PB = 0.90 WB = 0.87 CB = 0.88) DASS PEMS PES	N1 = 52.49 (16.71) N2 = 53.93 (18.42) N3 = 49.17 (16.63)	N1 = 39.67 (18.21) N2 = 42.81 (19.82) N3 = 37.69 (16.49)	N1 = 23.85 (20.30) N2 = 22.93 (19.87) N3 = 20.0 (15.72)	**Age** N1 (r = −0.15 ***) N2 (r = −0.21 ***) N3 (r = −0.14) **Years as midwife** N1 (r = −0.16 ***) N2 (r = −0.21 ***) N3 (r = −0.17) **Hours worked per week** N1 (r = 0.06) N2 (r = 0.14 ***) N3 (r = 0.22 ***) **Resource adequacy** *** N1 (r = −0.36) N2 (r = −0.46) N3 (r = −0.34) **Doctor/midwife relationships** *** N1 (r = −0.28) N2 (r = −0.25) N3 (r = −0.18) **Management support** *** N1 (r = −0.36) N2 (r = −0.43) N3(r = −0.24) **Autonomy and empowerment** N1 (r = −0.18 ***) N2 (r = −0.25 ***) N3 (r = −0.08)	2c	B
Fenwick et al. [35], 2018a, Australia	Cross-sectional Convenience sample N = 990	CBI	-	-	-	**Work area (continuity of care) (95% CI):** PB: OR = −0.92 (0.21–0.76) WB: OR = −0.86 (0.22–0.84) ** Having children (95% CI): PB: OR = −0.26 (0.49–1.23) WB: OR = −0.61 (0.34–0.85) CB: OR = −0.96 (0.18–0.82) **	2b	B
Fenwick et al. [44], 2018b, Australia	Cross-sectional Convenience sample N1 = 214 caseload midwives N2 = 648 standard care	CBI DASS PEMS	N1 = 50 N2 = 58.3	N1 = 35.7 N2 = 46.4	N1 = 8.3 N2 = 16.7	**Caseload care:** PB, WB, CB: Lowest rates versus non-continuity care (r = 0.11, r = 0.17 *, r = 0.11, respectively)	2c	B
Henriksen & Lukasse [41], 2016, Norway	Cross-sectional Random simple N = 598	CBI (PB = 0.89 WB = 0.89 CB = 0.90)	-	-	-	**Married/cohabitant (95% CI):** PB: OR = 0.6 (0.3–1.2) WB: OR = 0.5 (0.2–0.9) **No children (95% CI):** PB: OR = 1.2 (0.5–3.0) WB: OR = 1.3 (0.6–3.1) **Experience (<1 year) (95% CI):** PB: OR = 1.1 (0.7–2.5) WB: OR = 0.7 (0.3–1.4)	2c	B
Hildingsson et al. [42], 2013, Sweden	Cross-sectional Convenience sample N = 475	CBI (PB = 0.87 WB = 0.93 CB = 0.81)	42.99 (18.10)	33.85 (14.12)	30.42 (16.13)	**Conflicts with workmates and managers (95% IC):** PB: OR = 2.6 (1.4–5.1) ** CB: OR = 2.7 (1.2–5.7) **Lack of staff and resources (95% IC):** PB: OR = 2.1 (1.2–3.8) WB: OR = 3.9 (2.0–7.4) * CB: OR = 3.0 (1.6–5.8) *	2c	B
Hunter et al. [43], 2019, UK	Cross-sectional Convenience sample N = 1997	CBI (PB = 0.92 WB = 0.88 CB = 0.92)	65.4	56.15	25.36	Less than 10 years’ experience and aged 40 and below, are associated with high levels of burnout	2c	B
Jepsen et al. [39], 2017, Denmark	Cross-sectional Random simple N = 50	CBI	37.6 (16.2)	35.0 (15.7)	26.5 (16.4)	Caseload midwifery model care reduces burnout levels in all three subscales	2c	B
Jordan et al. [36], 2013, Australia	Cross-sectional Convenience sample N = 58	CBI (PB = 0.90 WB = 0.76 CB = 0.92)	52.1 (17.60)	50.9 (14.66)	23.9 (17.63)	PB and WB correlates with age and being single	2c	B
Kristensen et al. [5], 2005, Denmark	Cross-sectional Convenience sample N = 41	CBI (PB = 0.87 WB = 0.87 CB = 0.85)	44.7	43.5	38.4	Midwives have the highest score in the personal burnout and client-burnout dimensions	2c	B
Newton et al. [37], 2014, ^a,b^ Australia	Cross-sectional Convenience sample N1 = 21 caseload midwives N2 = 130 standard care	CBI (PB = 0.87 WB = 0.87 CB = 0.85) MPQ	N1 = 44.2 (21.2) N2 = 50.1 (17.5)	N1 = 41.1 (21.6) N2 = 45.1 (18.5)	N1 = 12.3 (9.6) N2 = 22.4 (18.0)	Caseload midwives have a higher level of job satisfaction. The positive aspects were: Continuity and relationships with known women, flexibility, autonomy	2c	B
Sidebotham et al. [38], 2015, Australia	Cross-sectional Convenience sample N = 1037	CBI DASS	55.9 (18.05)	48.44 (17.40)	25.59 (18.33)	One-third of midwives had moderate-high levels of anxiety and stress	2c	B
Stoll & Gallagher [40], 2018, Canada	Cross-sectional Convenience sample N = 136	CBI (PB = 0.90 WB = 0.89 CB = 0.91) DASS QOLS PEMS PES	60.4	46.8	28.5	The stressors found were: Workload and not enough time (64.6%), conflicts with workmates (42.4%), lack of care (39.9%), and difficulties in spontaneous labour support (35.4%)	2c	B

^a,b^ Two samples were present; * *p* < 0.001; ** *p* < 0.01; *** *p* < 0.05. Note: CB: Client-related burnout; CBI: Copenhagen Burnout Inventory; CS: Compassion satisfaction; DASS: Depression, Anxiety and Stress Scale; EL: Evidence level; QOLS: Quality of Life; MPQ: Midwifery Process Questionnaire; PB: Personal burnout; PEMS: Perceptions of empowerment in Midwifery Scale; PES: Practice Environment Scale; RG: Recommendation grade; WB: Work-related burnout.

**Table 2:** Criteria for assessing risk of bias for observational studies by Sanderson et al. [29].

Author	Selection Bias Sampling Source and Methods, with Inclusion/Exclusion Criteria	Measurement Bias Exposure and/or Outcome Measurement	Design Specific Bias Attrition Recall	Confounding Bias	Statistical Method Bias Primary Analysis of Effect	Conflict of Interest or Funding Source
Creedy et al. [32]	H	UC	L	L	L	H
Dawson et al. [33]	H	H	L	L	L	H
Dixon et al. [34]	H	H	L	L	L	H
Fenwick et al. [35]	H	UC	L	L	UC	H
Fenwick et al. [44]	H	H	L	L	L	L
Henriksen & Lukasse [41]	H	H	L	UC	UC	H
Hildingsson et al. [42]	H	H	L	L	L	L
Hunter et al. [43]	H	H	H	L	L	H
Jepsen et al. [39]	H	H	L	UC	L	L
Jordan et al. [36]	H	H	L	L	L	L
Kristensen et al. [5]	H	L	L	L	L	H
Newton et al. [37]	H	H	UC	L	UC	H
Sidebotham et al. [38]	H	L	L	L	L	L
Stoll & Gallagher [40]	H	H	L	L	L	UC

Note: H: High; L: Low; UC: Unclear.

**Table 3:** Prevalence of personal, work, and client related burnout (CBI scores > 50 points).

Author, Year	*n*	PB%	WB%	CB%
Creedy et al. [32], 2017	990	64.9	43.5	10.4
Dawson et al. [33], 2018	501	41	39	5
Fenwick et al. [35], 2018a	990	64.3	43.8	10.4
Henriksen & Lukasse [41], 2016	598	20.1	19.1	4.2
Hildingsson et al. [42], 2013	475	39.5	40	15
Hunter et al. [43], 2019	1997	82.8	67.4	15.5
Jepsen et al. [39], 2017	50	22	20	10
Jordan et al. [36], 2013	58	57	57	9
Newton et al. [37], 2014	N1 = 21	N1 = 35	N1 = 35	N1 = 0
	N2 = 130	N2 = 59	N2 = 46	N2 = 8
Stoll & Gallagher [40], 2018	136	74.9	45.2	20.3

## Supplementary Materials

The following are available online at https://www.mdpi.com/1660-4601/17/2/641/s1. Table S1: PRISMA checklist.
